# Endoscopic ultrasound–guided gastroenterostomy for the treatment of malignant gastric outlet obstruction in a 3-year-old boy

**DOI:** 10.1016/j.vgie.2026.02.012

**Published:** 2026-03-03

**Authors:** Timothy Adam, Michiel Bronswijk, Karen Pauwelyn, Schalk Van der Merwe

**Affiliations:** 1 Department of Gastroenterology and Hepatology, University Hospital Gasthuisberg, University of Leuven, Leuven, Belgium; 2 Department of Gastroenterology and Hepatology, Imelda Hospital Bonheiden, Bonheiden, Belgium; 3 Department of Gastroenterology and Hepatology, Noorderhart Hospital Pelt, Pelt, Belgium

## Abstract

**Background and Aims:**

Malignant gastric outlet obstruction (GOO) is an exceptionally rare and devastating condition in children, for which evidence-based management strategies are lacking. Although recent European guidelines endorse EUS-guided gastroenterostomy (EUS-GE) as an alternative to endoscopic stent placement and surgical gastroenterostomy in adults, its use in pediatrics is virtually unreported, to our knowledge. This absence of data leaves clinicians facing complex decision-making in profoundly challenging circumstances. We report on a pediatric case to illustrate the technical feasibility and potential clinical role of EUS-GE in malignant GOO.

**Methods:**

We describe stent placement during EUS-GE using the wireless EUS-GE simplified technique (WEST) in a 3-year-old boy with malignant GOO caused by recurrent abdominal neuroblastoma.

**Results:**

The procedure was technically and clinically successful, permitting resumption of oral intake and durable symptom control. The patient died 2 months later during palliative chemotherapy, without recurrence of GOO.

**Conclusions:**

This case highlights EUS-GE as a feasible palliative option for malignant GOO in highly selected pediatric patients. Given the rarity and severity of this condition, EUS-GE may be considered in expert centers with substantial experience in therapeutic EUS, underscoring the need for further reporting to inform future pediatric practice.

## Introduction

**Figure 1:**
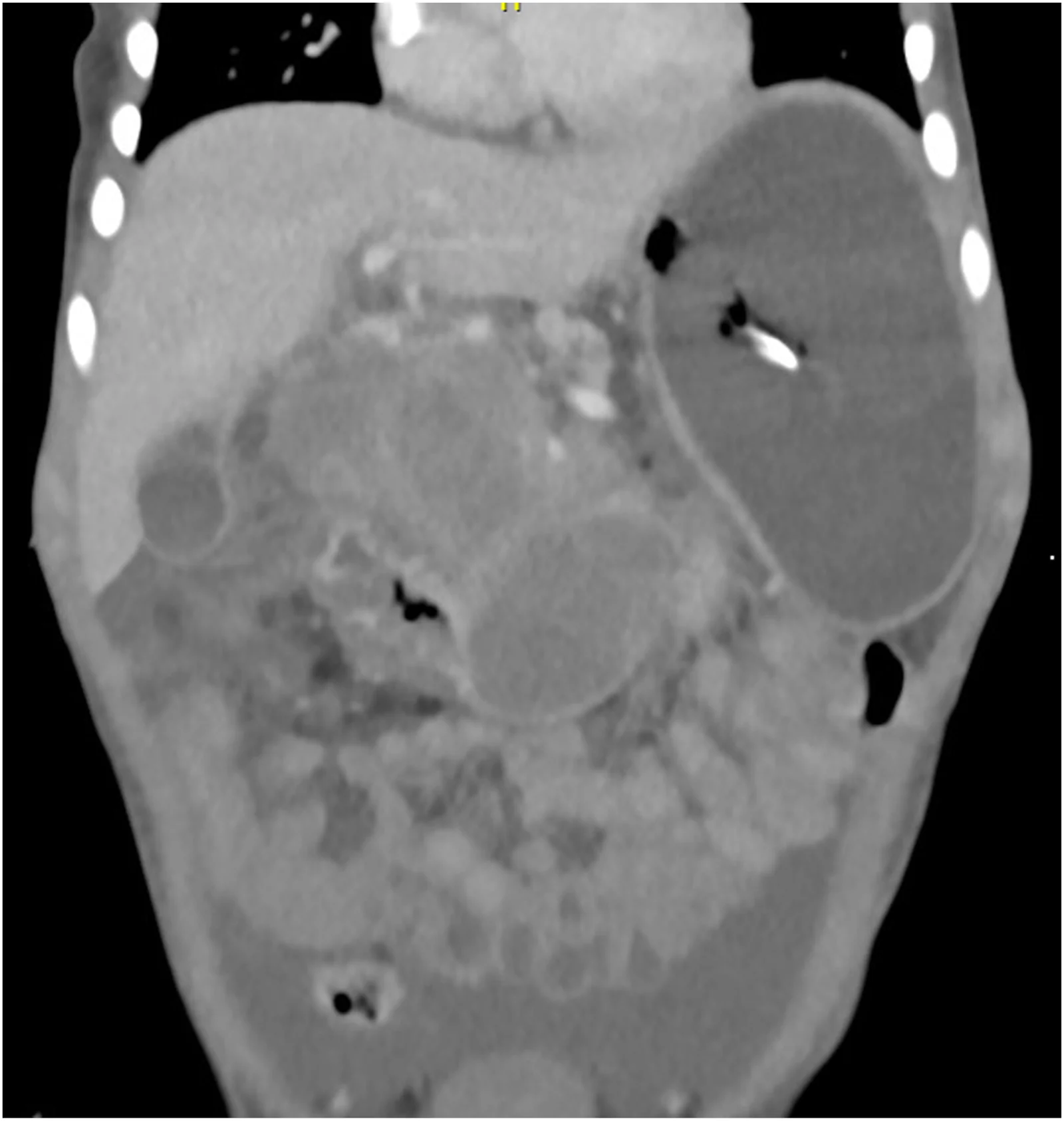
CT image showing gastric outlet obstruction secondary to the compression and invasion of the third part of the duodenum by the neuroblastoma.

A 3-year-old boy with a history of stage 4 abdominal neuroblastoma, diagnosed at the age of 2 years and treated under the high-risk neuroblastoma 2.0 protocol, presented to the department of pediatric oncology at our tertiary care center (Pediatric Oncology Department of the University Hospital Leuven, Leuven, Belgium) with persistent nausea and vomiting. At the time of referral to our department, the child had a body weight of 12.5 kg. CT imaging revealed a large tumor encasing the aorta, celiac trunk, and superior mesenteric artery, invading the duodenum and causing gastric outlet obstruction (GOO) (Fig. 1). After multidisciplinary discussion, given the prior extensive surgery, radiation, and palliative context, surgical intervention was deemed unsuitable. An EUS-guided gastroenterostomy (EUS-GE) was planned to palliate the obstruction.

## Procedure

With the patient under general anesthesia with protective intubation in supine positioning, the procedure was initiated by inserting a conventional gastroscope (GIF-HQ190; Olympus, Tokyo, Japan) into the second part of the duodenum, where tumoral invasion causing obstruction was visualized. A guidewire (VisiGlide, 0.025 in; Olympus) and a diagnostic catheter (MTW Endoskopie, Wesel, Germany) were carefully navigated past the obstruction up to the ligament of Treitz (Video 1, available online at www.videogie.org). The diagnostic catheter was then exchanged over the guidewire for a 7F nasobiliary catheter (MTW Endoskopie).

**Figure 2:**
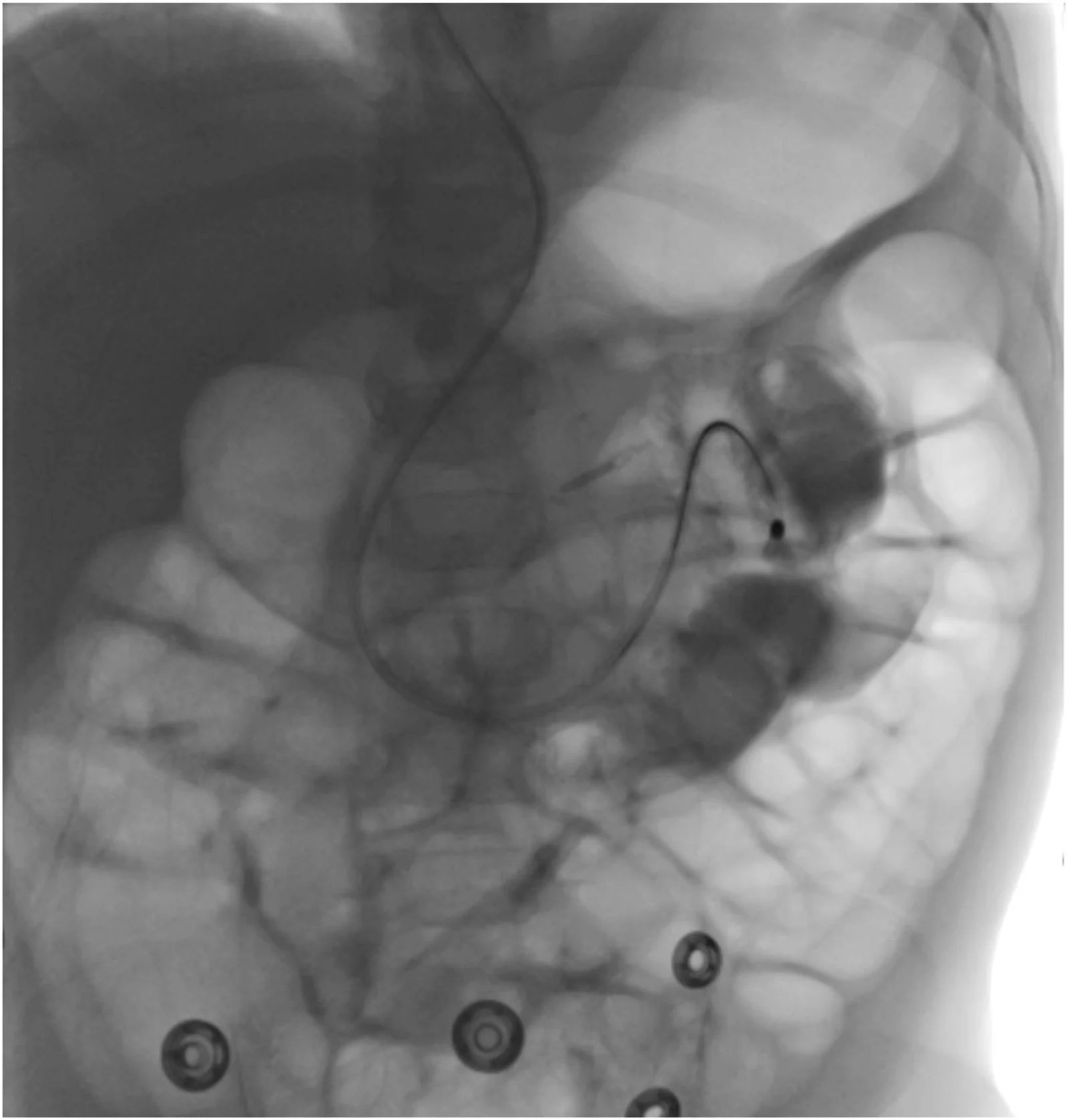
Fluoroscopy image after placement of the nasobiliary catheter passed the gastric outlet obstruction in the third part of the duodenum.

**Figure 3:**
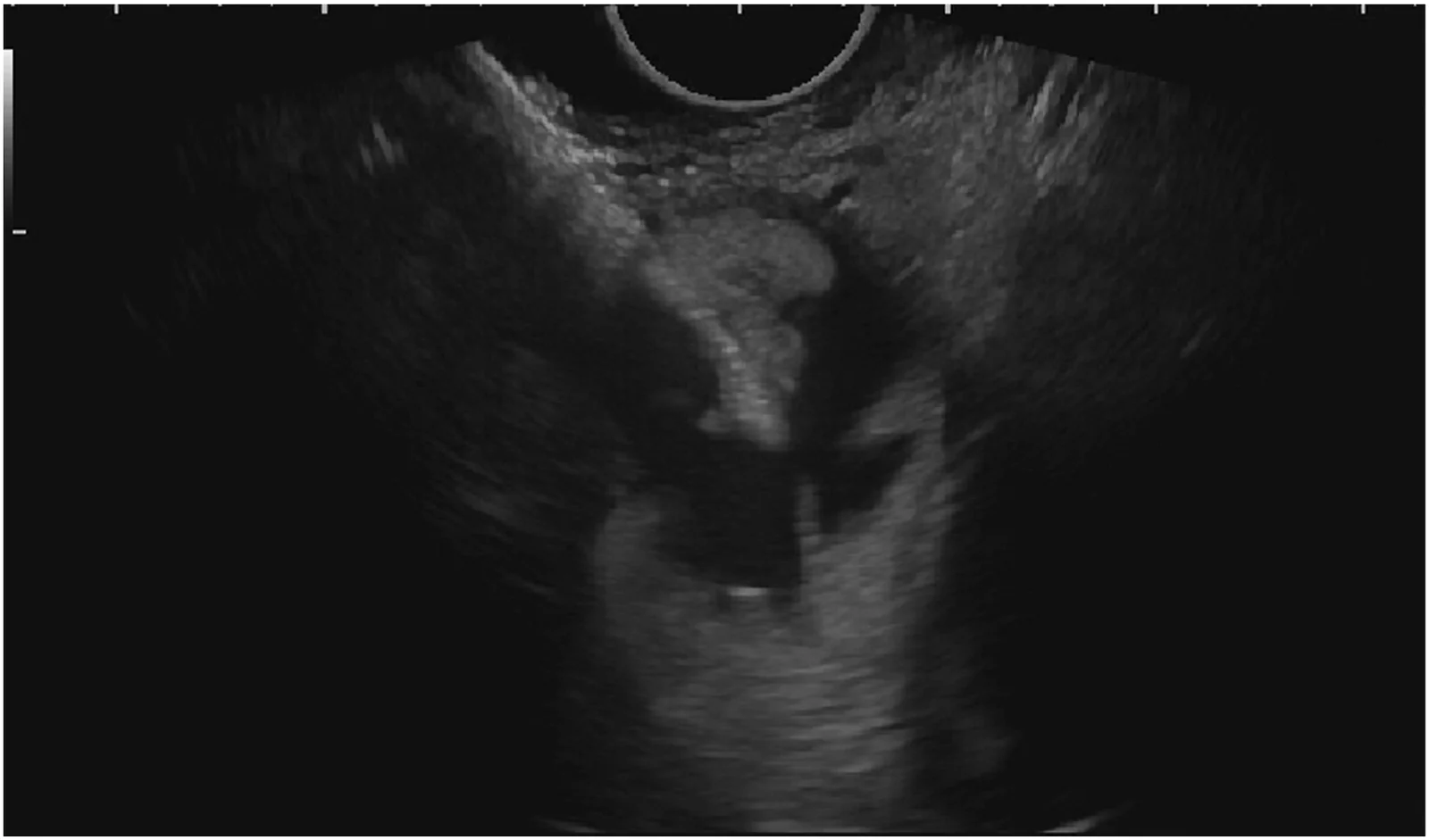
EUS-guided puncture of the small-bowel loop with the electrocautery-enhanced lumen-apposing metal stent. The smoke sign can be seen once the electrocautery-enhanced lumen-apposing metal stent passes into the small bowel.

**Figure 4:**
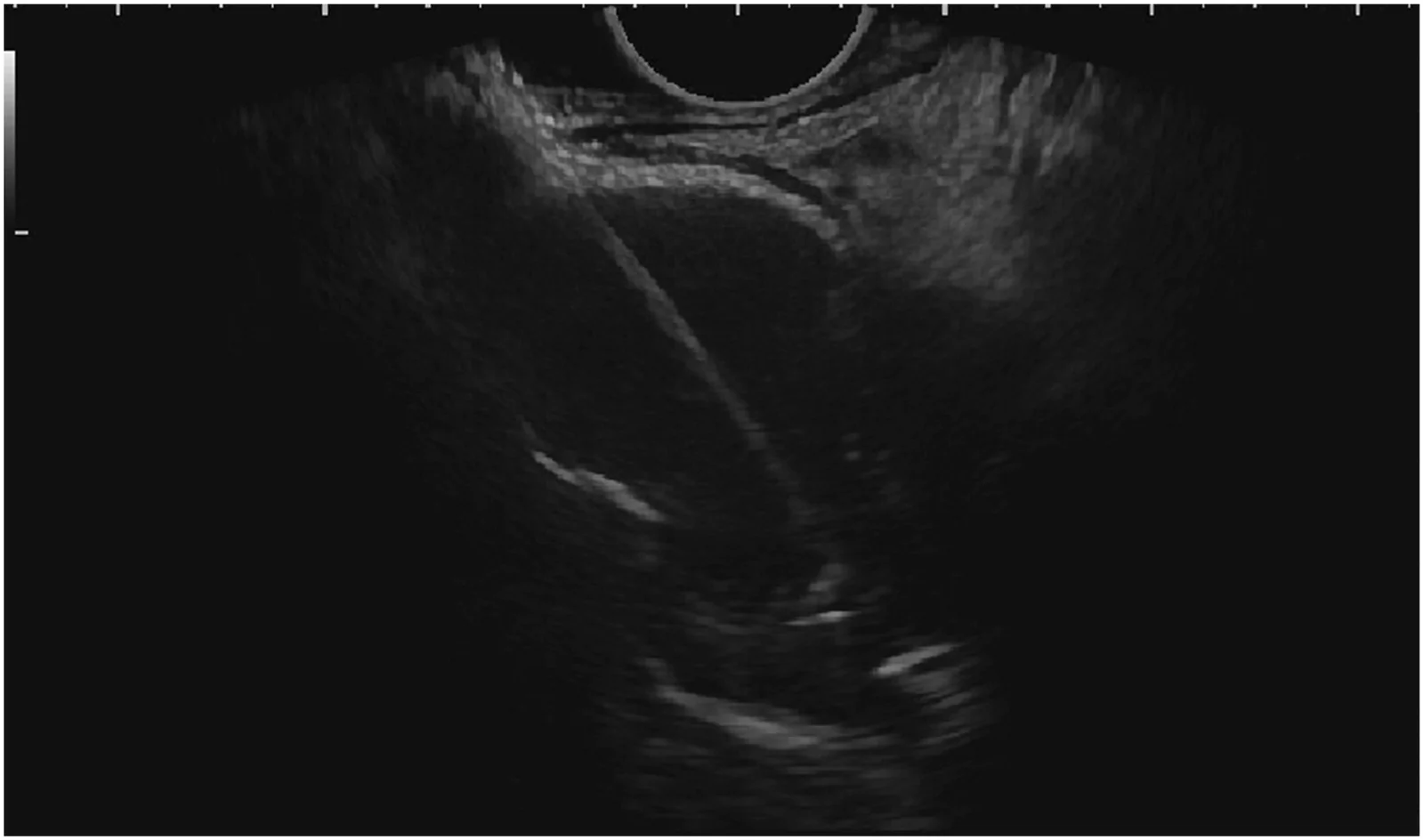
EUS-guided deployment of the distal flange of the lumen-apposing metal stent in the small bowel.

**Figure 5:**
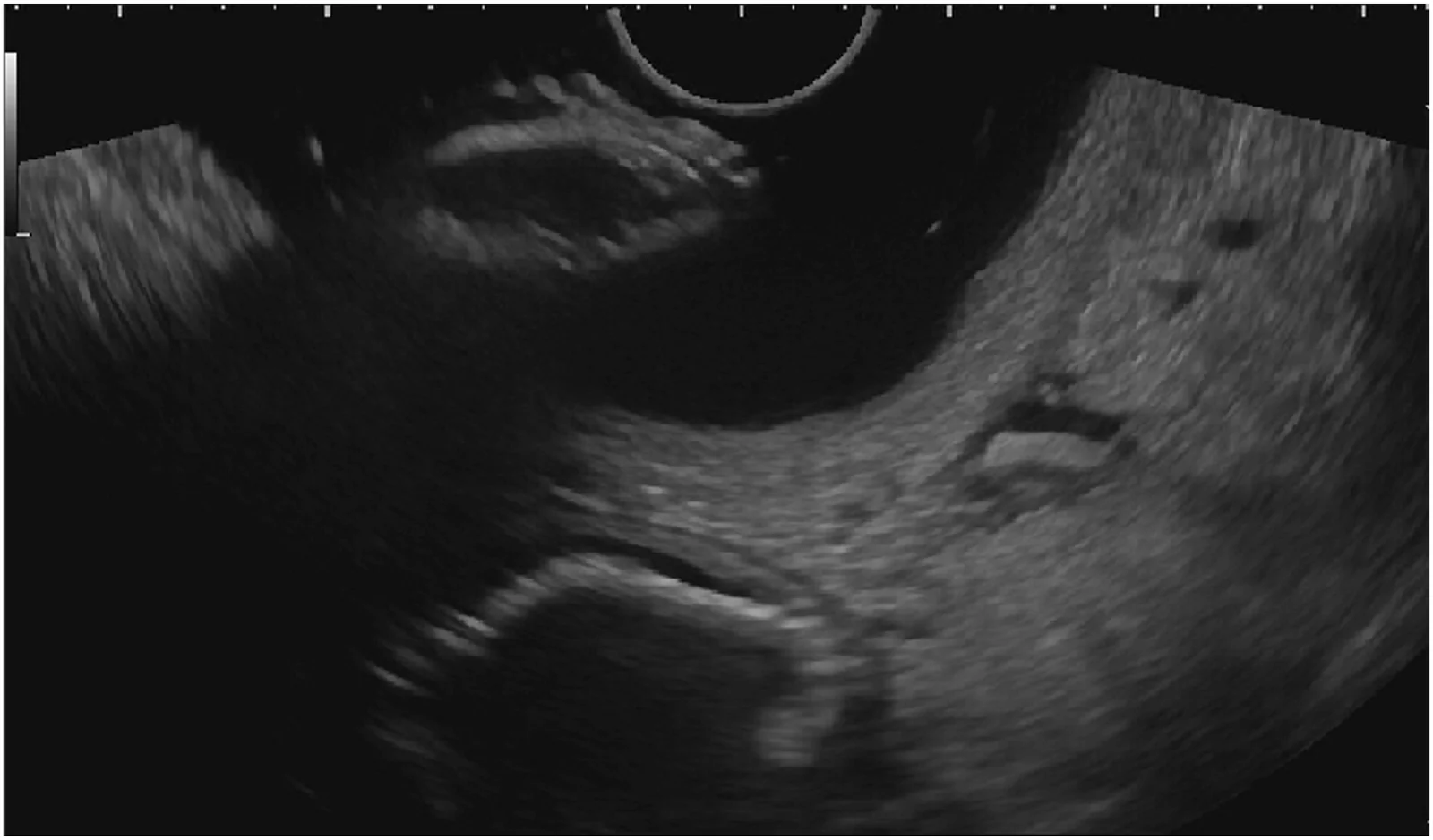
EUS image after deployment of the proximal flange of the lumen-apposing metal stent in the stomach.

**Figure 6:**
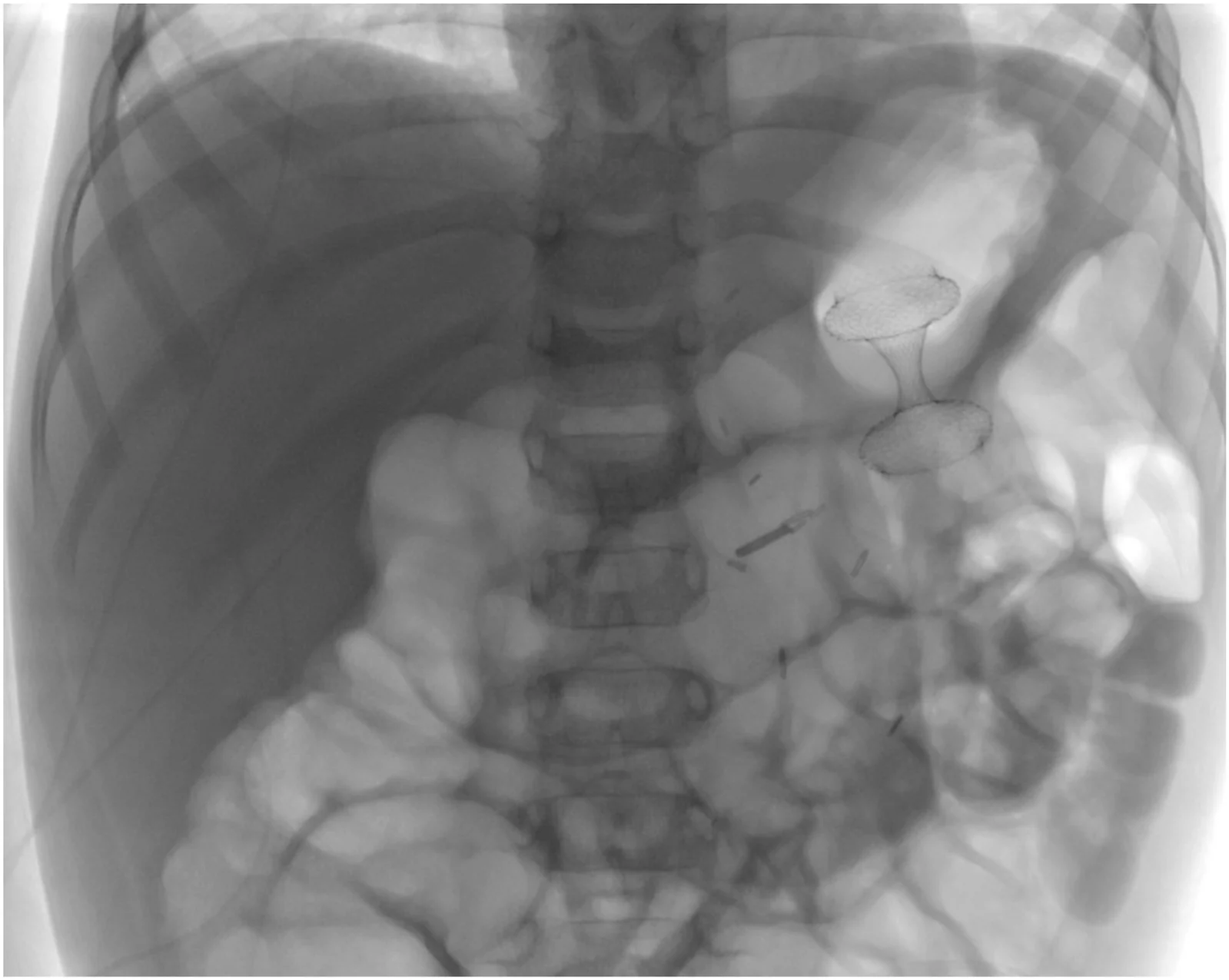
Fluoroscopic image after the placement of the lumen-apposing metal stent between the stomach and small bowel, creating a gastroenterostomy bypassing the gastric outlet obstruction.

**Figure 7:**
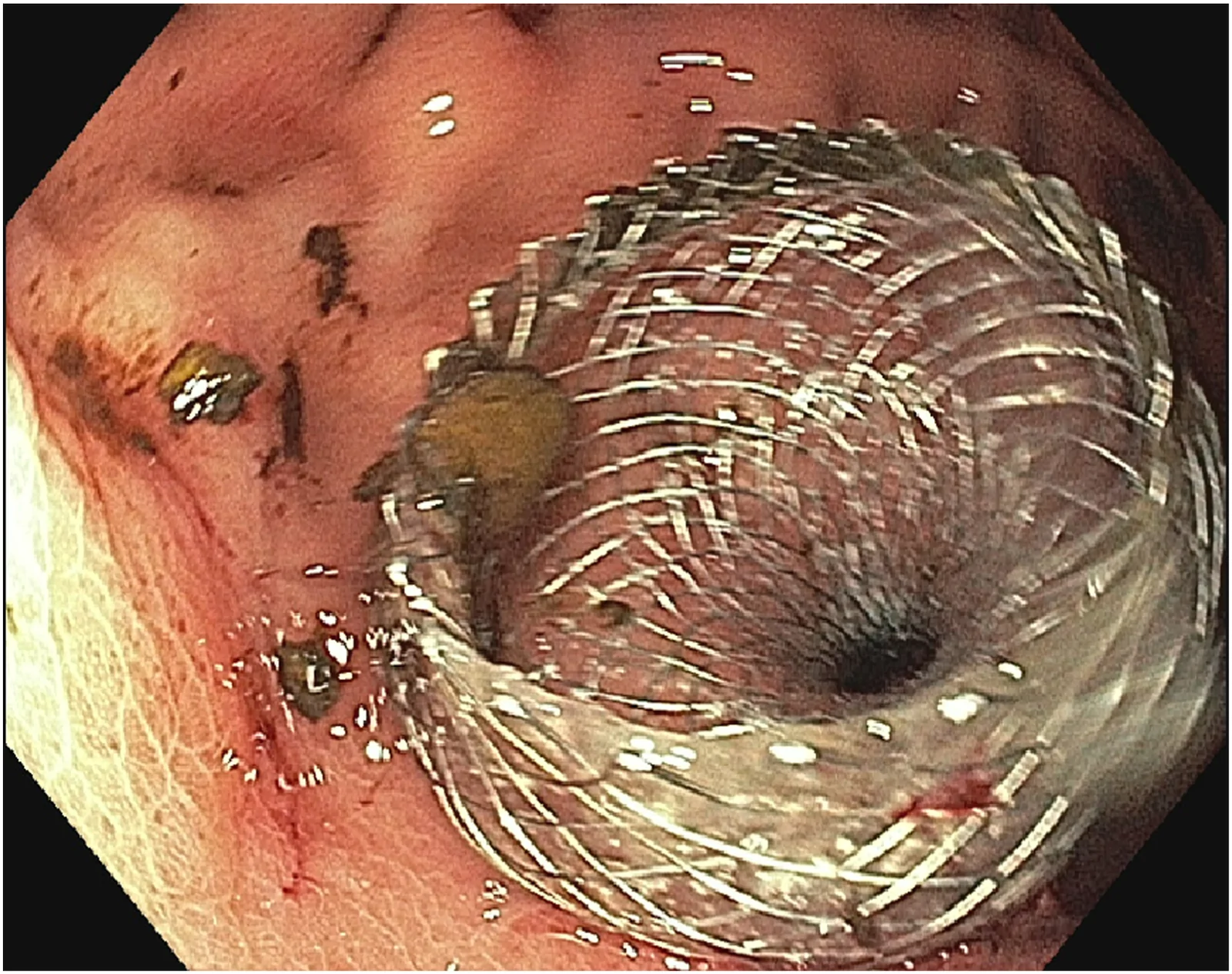
Endoscopic image of the proximal flange of the lumen-apposing metal stent in the stomach after deployment.

The gastroscope was subsequently removed, leaving the nasobiliary catheter in place (Fig. 2). A linear therapeutic EUS endoscope (EG38-J10UT; Pentax Medical, Tokyo, Japan) was then introduced alongside the catheter. Using combined EUS and fluoroscopic guidance, we identified an appropriate jejunal loop after saline solution instillation. No dye or antispasmodics were required in this case. Given the patient's body proportions, a 15- × 10-mm electrocautery-enhanced lumen-apposing metal stent (LAMS) was selected (Hot AXIOS, Boston Scientific, Marlborough, Mass, USA), and an EUS-GE was performed using the wireless EUS-GE simplified technique (WEST). No guidewire was used for LAMS insertion. After successful insertion of the device using pure cutting current (ENDO CUT effect 5, 120 W) (Fig. 3), we deployed the distal flange under direct EUS visualization, after which the device was retracted, compressing the different layers (Fig. 4). Subsequently, the proximal flange was released inside the working channel and deployed under endosonographic (Fig. 5) and endoscopic visual control. Successful placement was confirmed by observing the inflow of blue dye and contrast into the stomach (Fig. 6) and direct endoscopic visualization of small-bowel mucosa (Fig. 7). The total procedure time was 44 minutes. No LAMS dilation was performed given the lack of added benefit on patient outcomes and potential risk of stent dislocation.^1^ CO_2_-based insufflation was used throughout the procedure, and a single dose of broad-spectrum antibiotics was administered during the procedure.^2^

After EUS-GE, the patient was monitored in the recovery unit and resumed oral fluids on the same day, with subsequent gradual progression to a normal diet and no recurrence of GOO. Despite further palliative chemotherapy, the patient died 2 months later.

## Discussion

GOO is quite rare in the pediatric population outside the context of infantile hypertrophic pyloric stenosis. Other benign causes may include peptic ulcer disease, Crohn's disease, antral webs, or caustic injuries. Malignant GOO in children, however, is almost unreported, and lacks established pediatric management guidelines, to our knowledge.^3^, ^4^, ^5^

In adults, malignant GOO is typically managed with endoscopic stent (ES) placement or surgical gastroenterostomy (SGE), both of which have similar clinical success rates. ES, however, allows quicker resumption of oral intake, shorter hospital stays, and fewer adverse events, whereas SGE results in fewer recurrent obstructions and lower reintervention rates.^6^

Although EUS-GE is strictly speaking an off-label indication, recent European guidelines have recognized it as an alternative to ES placement and SGE in experienced centers.^7^ A randomized controlled trial demonstrated a lower reintervention rate at 6 months for EUS-GE than for ES placement, with comparable adverse events and clinical outcomes,^8^ confirming earlier retrospective and prospective series.^9^, ^10^, ^11^ In addition, EUS-GE has shown similar success and reintervention rates to SGE but with fewer adverse events.^1^^,^^12^

Various EUS-GE techniques have been described, most commonly the direct guidewire technique and WEST.^13^ Although the endoscopy community is getting closer to a standardized approach to EUS-GE,^14^ the most optimal technique is still the subject of debate, although WEST has shown higher technical success and fewer adverse events when compared in retrospective studies.^15^

Diagnostic EUS procedures, using adult linear US endoscopes, are feasible in children older than 3 years or weighing at least 15 kg.^16^ For therapeutic procedures, such as the current, extreme care should be taken to prevent injury during device manipulation, given the disproportionate size of scopes and accessories. In this case report, to our knowledge, we provide the first evidence of the successful placement of an EUS-GE in a 3-year-old patient with malignant GOO using WEST. Therefore, EUS-GE can be considered in rare pediatric patients with malignant GOO in expert centers with extensive experience in therapeutic EUS.

## Disclosure

The following authors disclosed financial relationships: M. Bronswijk: Trial support from Boston Scientific; consultancy agreement for Ovesco Ag, Dekra, and Prion Medical. S. Van der Merwe: Chair in Interventional Endoscopy at Cook Medical; co-chair in Therapeutic Biliopancreatic Endoscopy at Boston Scientific; consultancy agreement for Cook Medical, Pentax, and Olympus. All other authors disclosed no financial relationships.

## Supplementary data

Video 1 Endoscopic ultrasound-guided gastroenterostomy in a 3–year–old boy
